# Pharmacodynamic and pharmacokinetic assessment of pulmonary rehabilitation mixture for the treatment of pulmonary fibrosis

**DOI:** 10.1038/s41598-017-02774-1

**Published:** 2017-06-14

**Authors:** Juanjuan Zhao, Yan Ren, Yubei Qu, Wanglin Jiang, Changjun Lv

**Affiliations:** School of Pharmaceutical Sciences, Binzhou Medical University, Yantai, PR China

## Abstract

Pulmonary rehabilitation mixture (PRM), a Chinese herbal medicine formula, has been used to treat pulmonary fibrosis for decades. In this study, we systematically evaluated the pharmacodynamic and pharmacokinetic performance of PRM. The pharmacodynamic results showed that PRM could improve the condition of CoCl_2_-stimulated human type II alveolar epithelial cells, human pulmonary microvascular endothelial cells, human lung fibroblasts and pulmonary fibrosis rats induced by bleomycin, PRM treatment reduced the expression of platelet-derived growth factor, fibroblast growth factor, toll-like receptor 4, high-mobility group box protein 1 and hypoxia-inducible factor 1α. In the pharmacokinetic study, an accurate and sensitive ultra-high performance liquid chromatography tandem mass spectrometry method was developed and validated for the simultaneous determination of calycosin, calycosin-7-O-glucoside, formononetin, ononin and mangiferin of PRM in the rat plasma for the first time. The method was then successfully applied to the comparative pharmacokinetic study of PRM in normal and pulmonary fibrosis rats. The five constituents could be absorbed in the blood after the oral administration of PRM and exhibited different pharmacokinetic behaviors in normal and pulmonary fibrosis rats. In summary, PRM exhibited a satisfactory pharmacodynamic and pharmacokinetic performance, which highlights PRM as a potential multi-target oral drug for the treatment of pulmonary fibrosis.

## Introduction

Pulmonary fibrosis (PF) is a chronic, progressive and life-threatening fibrotic disease of the lung, which carries a median survival of three to four years after diagnosis^1^. The prevalence of PF is 2 to 29 per 100 000 persons and its incidence is approximately 10 per 100 000 persons per year, with an upward trend^2^. Unfortunately, there are no proven effective therapies for PF beyond lung transplantation, although pirfenidone has been approved as a novel antifibrotic agent in some countries^3^. Traditional therapy for PF has consisted of rednisone, azathioprine and N-acetylcysteine; however, patients who took prednisone, azathioprine and NAC (combination therapy), NAC alone or placebo suffered mild-to-moderate lung function impairments^4–6^. The pathogenesis and drug therapy of PF remain poorly elucidated and controversial.

In recent years, Chinese herbal prescriptions have attracted increasing attention due to their complementary therapeutic effects to western drugs but with minimum side effects. Chinese herbal prescriptions, which contain several crude drugs to obtain beneficial bioactivities for clinical indications, play an important role in the treatment of many complex diseases because Chinese herbal prescriptions are multi-target therapies^7, 8^.

Pulmonary rehabilitation mixture (PRM), also known as Fei-Fu-Kang (FFK), is a Chinese herbal medicine formula that is designed based on an empirical-based traditional Chinese medicine prescription. PRM has been used to treat patients with PF and lung cancer for several decades. Our research has demonstrated that PRM could prevent PF through modulating the high mobility group box 1 (HGMB1) and receptor for advanced glycation end-product (RAGE) pathways *in vitro* and *in vivo* ^9^. In addition, PRM treatment could attenuate lung tissue edema and histological changes in acute lung injury in mice induced by lipopolysaccharide through the inhibition of the MAPK signaling pathways. However, the mechanism of PRM on PF was not fully elaborated. Therefore, we further investigated the effects of PRM on experimental pulmonary fibrosis *in vitro* and *in vivo* and proposed a mechanism of action for PRM.

PRM is composed of eight herbs including *Astragali Radix, Codonopsis Radix, Ophiopogonis Radix, Schisandrae Chinensis Fructus, Notoginseng Radix et Rhizoma, Fritillariae Thunbergii Bulbus, Anemarrhenae Rhizoma and Glycyrrhizae Radix et Rhizoma. Astragali Radix*, the monarch drug of PRM, is reported to possess several medicinal functions including antitumor, immunostimulating and antiaging effects^10, 11^. In addition, the isoflavonoid compounds of PRM, such as calycosin, calycosin-7-O-glucoside, formononetin, ononin and mangiferin, are effective medicinal ingredients due to their pharmacological activities and therapeutic efficacies^12–15^.

However, to our knowledge, no pharmacokinetic study of PRM has been reported. The characterization of pharmacokinetics is essential for clarifying the mechanism of action and enabling instructions for the practical application of PRM. Thus, a selective and sensitive ultra-high performance liquid chromatography tandem mass spectrometry (U-HPLC-MS/MS) method was developed and validated for the simultaneous determination of calycosin, calycosin-7-O-glucoside, formononetin, ononin and mangiferin derived from PRM in rat plasma. The method was used to determine the pharmacokinetic profile of PRM, which could be helpful in the clinical use of PRM.

Therefore, we investigated the effects of PRM on experimental pulmonary fibrosis *in vitro* and *in vivo* and proposed a mechanism of action of PRM. In addition, the pharmacokinetics properties of calycosin, calycosin-7-O-glucoside, formononetin, ononin and mangiferin were systematically studied in normal and PF rats after the oral administration of PRM.

## Materials and Methods

### Cells

Human lung fibroblasts (HLF-1), Human Pulmonary Microvascular Endothelial Cells (HPMVECs) and Human type II alveolar epithelial cells (A549 cell line) were purchased from the Cell Bank of the Chinese Academy of Sciences. Cells were maintained in a Dulbecco’s modified Eagle’s medium (DMEM) containing 10% newborn calf serum, 100 U/mL penicillin and 100 mg/mL streptomycin at 37 °C in a humidified atmosphere of 5% CO_2_ and 95% N_2_. Cells were subcultured every 3–4 days when they reached a density of 1 × 10^5^/mL.

### Animals

Male pathogen-free Sprague-Dawley rats (180–200 g), provided by the Animal Center of Luye Pharma Group (Yantai, China), were housed individually at a constant temperature (22 ± 26 °C) and humidity with a 12-h light/dark cycle and free access to chow and water. The animal studies were carried out in accordance with the Institutional Animal Care and National Institutes of Health Guidelines for the Care and Use of Laboratory Animals (USA), and the protocol was approved by the Committee on the Ethics of Animal Experiments of Binzhou Medical University (Permit No. SCXK20140005).

### Chemicals and reagents

All of the herbs in PRM were obtained from Yantai Tongrentang Drug Company (Yantai, China) and authenticated with the Chinese Pharmacopeia 2010. Calycosin, calycosin-7-O-glucoside, formononetin, ononin and mangiferin (purity > 98%) were purchased from Shanghai Ronghe Biotechnology Co. Ltd (Shanghai, China). Sulfamethoxazole (IS) was provided by the National Institute for Food and Drug Control (Beijing, China). HPLC grade acetonitrile and formic acid were obtained from Fisher Scientific (Fair Lawn, NJ, USA) and Merck (Darmstadt, Germany), respectively. Ethyl acetate and isopropanol (HPLC grade) were acquired from Concord Tech. Co. (Tianjin, China). Purified water that was prepared with demineralized water was used throughout the study. HMGB1, hypoxia-inducible factor 1α (HIF-1α), fibroblast growth factor (FGF-2), platelet-derived growth factor (PDGF) and toll-like receptor 4 (TLR-4) in the study were all provided by Abcam Company (Cambridge, MA, USA).

### PRM extraction

**Figure 1:**
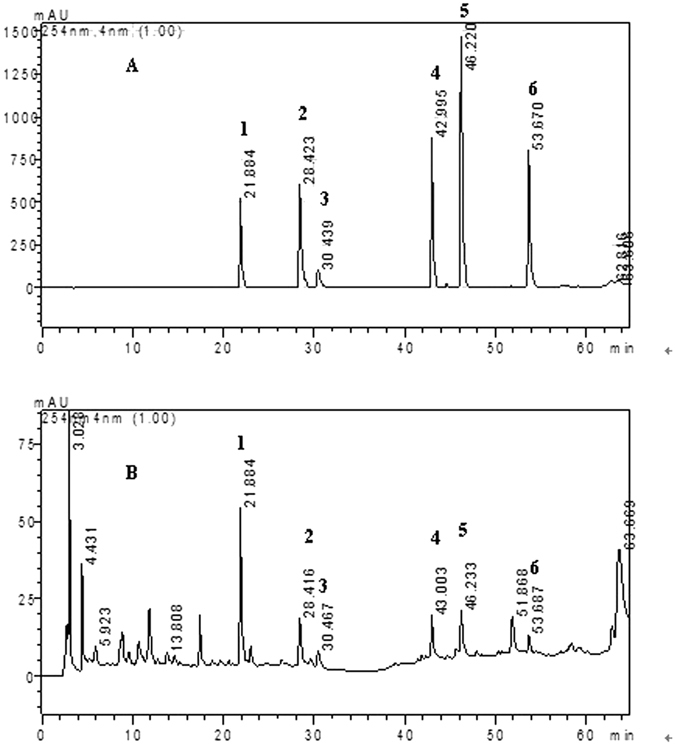
HPLC fingerprint of mix standards (**A**) and PRM (**B**). 1: mangiferin, 2: calycosin-7-O-glucoside, 3: liquiritin, 4: ononin, 5: calycosin, 6: formononetin.

*Astragali Radix* (1200 g), *Codonopsis Radix* (700 g), *Ophiopogonis Radix* (480 g), *Schisandrae Chinensis Fructus* (360 g), *Notoginseng Radix et Rhizoma* (240 g), *Fritillariae Thunbergii Bulbus* (240 g), *Anemarrhenae Rhizoma* (240 g) and *Glycyrrhizae Radix et Rhizoma* (120 g) were mixed and then extracted twice in 20 L of 50% ethanol for 24 h each time. The extracted solution was combined and evaporated to dryness under reduced pressure. Then, the crude extract was reconstituted with 10 L distilled water. The solution was then centrifuged at 3000 g for 15 min at 4 °C, and the supernatant was collected as PRM. The concentration of PRM was 0.5 g crude drug per milliliter. To control the quality of PRM, its fingerprint was prepared by high performance liquid chromatographic method (HPLC) at 254 nm and is shown in Fig. 1. The content of Calycosin, calycosin-7-O-glucoside, formononetin, ononin and mangiferin in PRM was quantitatively analyzed. The content was 124.4 µg/g of calycosin, 220.4 µg/g of calycosin-7-O-glucoside, 50.8 µg/g of formononetin, 173.6 µg/g of ononin, and 296.7 µg/g of mangiferin.

### Experimental model of pulmonary fibrosis by Bleomycin (BLM)

The male SD rats (180–220 g) were first anaesthetized by an intraperitoneal injection of 0.5 mg/kg ketamine and 1 mg/kg xylazine; then, the proximal portion of trachea was exposed, and bleomycin hydrochloride (6 mg/kg) in 0.3 mL saline was administered by a single intratracheal instillation to induce pulmonary fibrosis *in vivo* ^16^. The control rats were treated with an equal volume of saline during the same procedure.

## Pharmacodynamic experiment

### Effect of PRM on pulmonary fibrosis *in vitro*

A549 cells and HPMVECs were maintained in DMEM/F12 and DMEM (high glucose), respectively, both of which contained 10% (v/v) fetal bovine serum, 100 kU/L penicillin and 100 mg/L streptomycin at 37 °C in a humidified 5% CO_2_ atmosphere. The cells were cultured to approximately 70% confluency, starved in serum-free DMEM overnight and treated with PRM at a concentration of 0.3 mg/mL with or without 100 μM of CoCl_2_ for 48 h. Images of five random fields at a 400x magnification were taken using an inverted microscope to investigate the mechanism of the effects of PRM on cell morphology. HMGB1, HIF-1α, vimentin, E-cadherin and VE-cadherin expression in CoCl_2_-stimulated A549 cells and HPMVECs were analyzed by western blots to investigate the mechanism of PRM on epithelial-mesenchymal transition (EMT) and endothelial-mesenchymal transition (EndoMT). The expression of these proteins was normalized to the expression of β-actin. The results were expressed as the fold increase over the normal.

For the proliferation assays *in vitro*, HLF-1 cells were inoculated into 96-well (1 × 10^5^ cells/well) flat bottom plates with medium alone (control) or with a medium containing PRM (0.3 mg/mL) with or without 100 μM CoCl_2_. Cell proliferation was assessed using a methythiazol tetrazolium (MTT) assay. The absorbance was recorded at 490 nm (Spectramax/M5 multi-detection reader, Molecular Devices, USA) and is presented as a ratio compared to untreated cells. To investigate the mechanism of PRM on cell proliferation, the expression levels of FGF-2, PDGF-BB, HMGB1 and HIF-1α in CoCl_2_-stimulated HLF-1 cells were analyzed by western blots. The expression of these proteins was normalized against the expression of β-actin. The results were expressed as the fold increase over the normal.

### Effects of PRM on pulmonary fibrosis *in vivo*

Thirty male SD rats (180–220 g) were acclimatized for 7 days and then randomly divided into three groups, including the control group, the PF group and the PRM group. Rats in the PF group and the PRM group were both treated with bleomycin hydrochloride (6 mg/kg) to induce pulmonary fibrosis as described above. Beginning on day 28, rats in the PRM group rats received oral PRM 3 g/kg daily. The lung was removed on day 42 and divided into the following two parts: one part was frozen in liquid nitrogen, and the other was fixed in 10% formalin for further analysis.

Lung samples were suspended in a buffer that contained 10 mM Tris (pH 7.5), 1.5 mM MgCl_2_, 10 mM KCl, and 0.1% Triton X-100 and lysed by homogenization; the supernatant was collected and stored at −80 °C for western blot analysis. The protein concentration was measured by a bicinchoninic acid (BCA) assay. After the determination of the protein concentration, equal amounts of the protein (approximately 50 µg) were isolated by SDS-PAGE and transferred to a PDVF membrane. Then, the membrane was blocked with TBST containing 5% skim milk power. The expression levels of a variety of proteins were analyzed by western blot using specific antibodies against PDGF, FGF-2, TLR-4, HMGB1, HIF-1α and β-actin. The optical densities of the bands were scanned and quantified with a Gel Doc 2000. The densities were normalized against those of the corresponding β-actin band. The results are presented as percentage increase compared to the values form the sham treated rat.

## Pharmacokinetic experiment

### Chromatographic and mass spectrometric conditions

The samples were analyzed using a Thermo Accela 1250 U-HPLC system coupled to a TSQ Quantum Access MAX mass spectrometer (Thermo Fisher Scientific, Waltham, MA, USA). The U-HPLC system consisted of a quaternary pump, an autosampler and a column oven. The chromatographic separation was performed on a Sunfire C_18_ column (150 mm × 2.1 mm i.d., 3.5 μm particle size) maintained at 35 °C. The mobile phase, pumped at a flow rate of 0.2 mL/min, consisted of acetonitrile (A) and 0.1% formic acid in water (B). The initial mobile phase composition was 30% A/70% B. After the sample injection, the mobile phase composition was changed linearly to 90% A/10% B over 8.0 min, was held constant for an additional 3.0 min, and then returned to 30% A/70% B immediately for re-equilibration. The total analysis time was 12 min. The autosampler was conditioned at 4 °C and the injection volume was 5 μl.

The applied TSQ Quantum Access MAX mass spectrometer was operated in the multiple reactions monitoring (MRM) mode by an electrospray ionization interface. Ionization was conducted in the negative ion mode for mangiferin and the positive ion mode for the other analytes. The quantitative parameters of the analytes are summarized in Table 1. The parameters of the mass spectrometer were set as follows: spray voltage at 3000 V, vaporizer temperature at 372 °C, capillary temperature at 300 °C, aux gas pressure at 10 arb, and collision pressure at 1.0 arb.

**Table 1:** The main quantitative parameters of MRM for calycosin, calycosin-7-O-glucoside, formononetin, ononin and mangiferin.

Analyte	Q1 Mass (Da)	Q3 Mass (Da)	Tube Lens	Collision energy
Calycosin	285.0	270.0	84	22
		213.0	84	36
Calycosin-7-O-glucoside	447.1	285.0	64	18
		269.9	64	36
Formononetin	269.0	197.0	81	40
		253.0	81	29
Ononin	431.1	269.0	65	18
		252.8	65	48
Mangiferin	421.0	300.9	68	26
		330.9	68	23
Sulfamethoxazole	254.0	156.1	63	15
		108.3	63	21

### Preparation of standard solution and quality control samples

Stock solutions of calycosin, calycosin-7-O-glucoside, formononetin, ononin and mangiferin were prepared at concentrations of 0.04 mg/mL in methanol, respectively. Mixed working standard solutions were obtained by serial dilution in methanol. In addition, a stock solution of sulfamethoxazole (IS) was prepared in methanol and subsequently diluted with methanol to 600 ng/mL as a working solution. All of the solutions were stored at 4 °C.

Calibration samples at concentrations of 0.2, 0.5, 1, 2, 10, and 50 ng/mL for calycosin, calycosin-7-O-glucoside, formononetin, ononin and mangiferin were prepared by spiking the appropriate amount of a working standard solution in blank rat plasma. Three levels of quality control (QC) samples (0.5, 5 and 40 ng/mL for all of the analytes except IS) in plasma were prepared separately in the same fashion.

### Sample preparation

Plasma samples (200 μl) were spiked with 20 μl of IS, 20 μl of methanol and 50 μl of 0.25 M hydrochloric acid; vortexed for 1 min; and then extracted with 1 mL ethyl acetate-isopropanol (7:3, v/v) by vortexing for 5 min. After centrifugation at 1083 × *g* for 5 min, the supernatant was transferred to another centrifuge tube and evaporated to dryness at 35 °C under a slight stream of nitrogen. Then the residue was reconstituted with 100 μl of the initial mobile phase by vortexing for 1 min. Finally, 5 μl of the supernatant that was obtained after centrifugation at 1083 × *g* for 5 min were used for the analysis.

### Method validation

The method was fully validated according to the “Guidance for Industry: Bioanalytical Method Validation” approved by the US Food and Drug Administration (FDA) with respect to specificity, matrix effect, linearity, accuracy, precision, recovery and stability^17^.

### Pharmacokinetic applications

Twelve male SD rats (180–220 g) were acclimatized for 7 days and then randomly divided into two groups, the control group and the PF group. The rats in the PF group were treated with bleomycin hydrochloride (6 mg/kg) to induce pulmonary fibrosis as described above. On the 28^th^ day, the rats in both groups received oral PRM at a dose of 3 g/kg. Then, blood was collected from the suborbital vein before the administration of PRM and 0.08, 0.17, 0.33, 0.5, 0.75, 1, 1.5, 2, 3, 4, 6, 8, 12 and 24 h after treatment and was immediately centrifuged at 4650 × *g* for 5 min. The harvested plasma samples were stored at −20 °C and analyzed within a week.

## Results and Discussion

### Effects of PRM on pulmonary fibrosis *in vitro*

#### Effects of PRM on EMT in A549 cells

**Figure 2:**
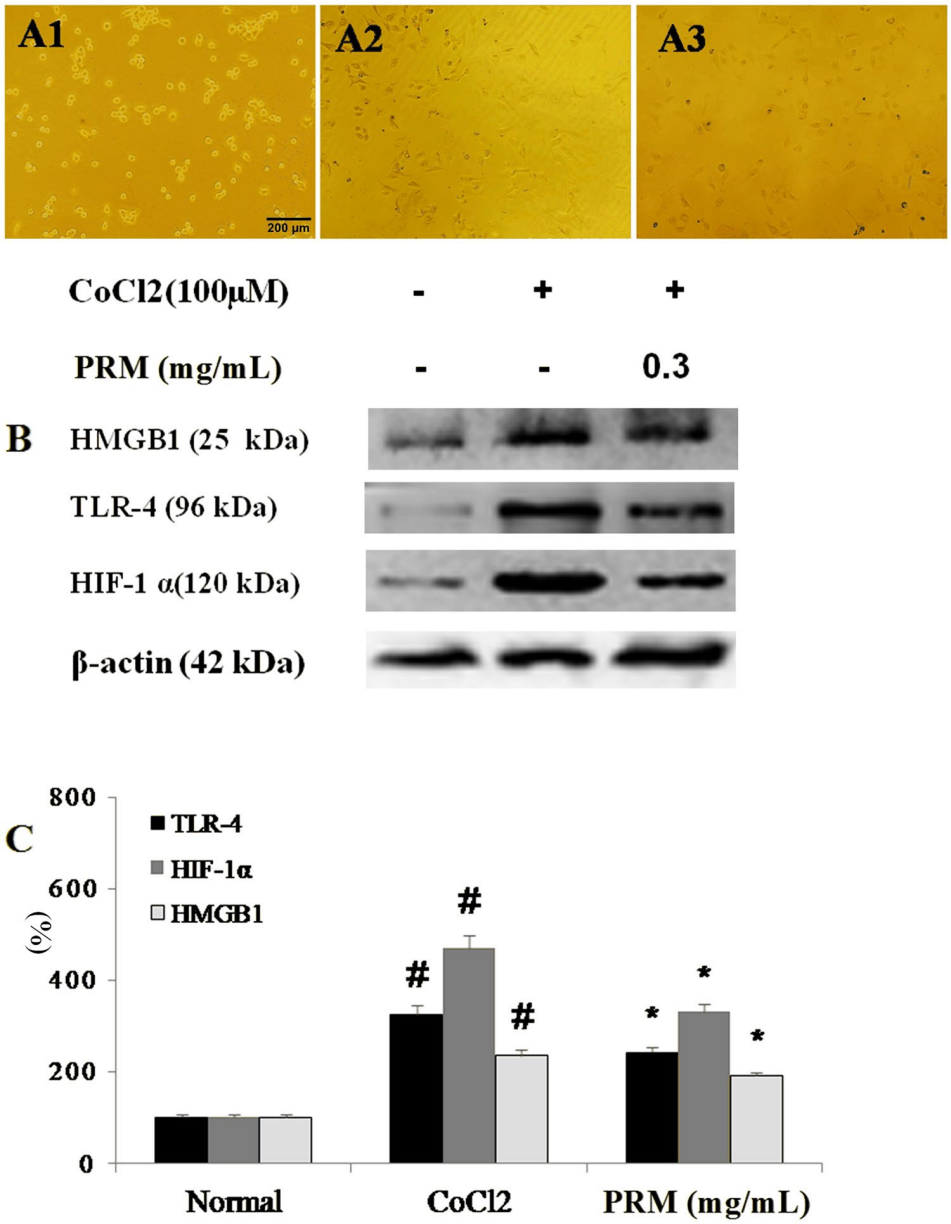
Effects of PRM on EMT in A549 cells. (**A**) Representative light microscopic appearance of A549 cells of normal A549 cells (A1), CoCl_2_-treated A549 cells (A2), and cells treated with CoCl_2_ +0.3 mg/mL PRM (A3). (**B,C**) A549 cells were incubated with CoCl_2_ (100 μM) for 48 h. HMGB1, TLR-4 and HIF-1α expression was analyzed by western blot. The results are reported as percent increase over the normal. Data are reported as the mean ± S.D., n = 5. ^#^P < 0.01 vs. the normal group; ^*^P < 0.05, ^**^P < 0.01 *vs*. the CoCl_2_ stimulated group. Significance was determined by one-way ANOVA followed by Dunnett’s test. The full-length blots from Fig. 2B are presented in Supplementary information 2.1.

EMT was significantly enhanced in A549 cells that were treated with 100 μM CoCl_2_ for 48 h; EMT was noticeably attenuated in the CoCl_2_-stimulated cells that were exposed to 0.3 mg/mL PRM as shown in Fig. 2A. To investigate how PRM might reduce EMT, its effects on the expression of the epithelial cell marker E-cadherin and the mesenchymal cell marker vimentin were analyzed by western blot. The results indicated that the PRM treatment of CoCl_2_-stimulated A549 cells attenuated the increase in the expression of HMGB1, TLR-4 and HIF-1α as shown in Fig. 2B and C.

Lung fibrotic pathogenesis is driven by abnormally activated alveolar epithelial cells (AECs). AECs promote EMT and the differentiation of fibroblasts into myofibroblasts via the activation of hypoxia. The epithelial-dependent fibroblast activation process was found in case of experimental and clinical pulmonary fibrosis. During EMT, TLR-4, HMGB1 and HIF-1α levels are increased^18–20^. The results indicated PRM treatment could result in an EMT phenotypic reversal and the normalization of TLR-4, HMGB1 and HIF-1α expression in A549 cells. Therefore, A549 cells treated with PRM were resistant to CoCl_2_-induced EMT, whereas PRM-untreated A549 cells were more vulnerable to EMT.

#### Effects of PRM on EndoMT in HPMECs

**Figure 3:**
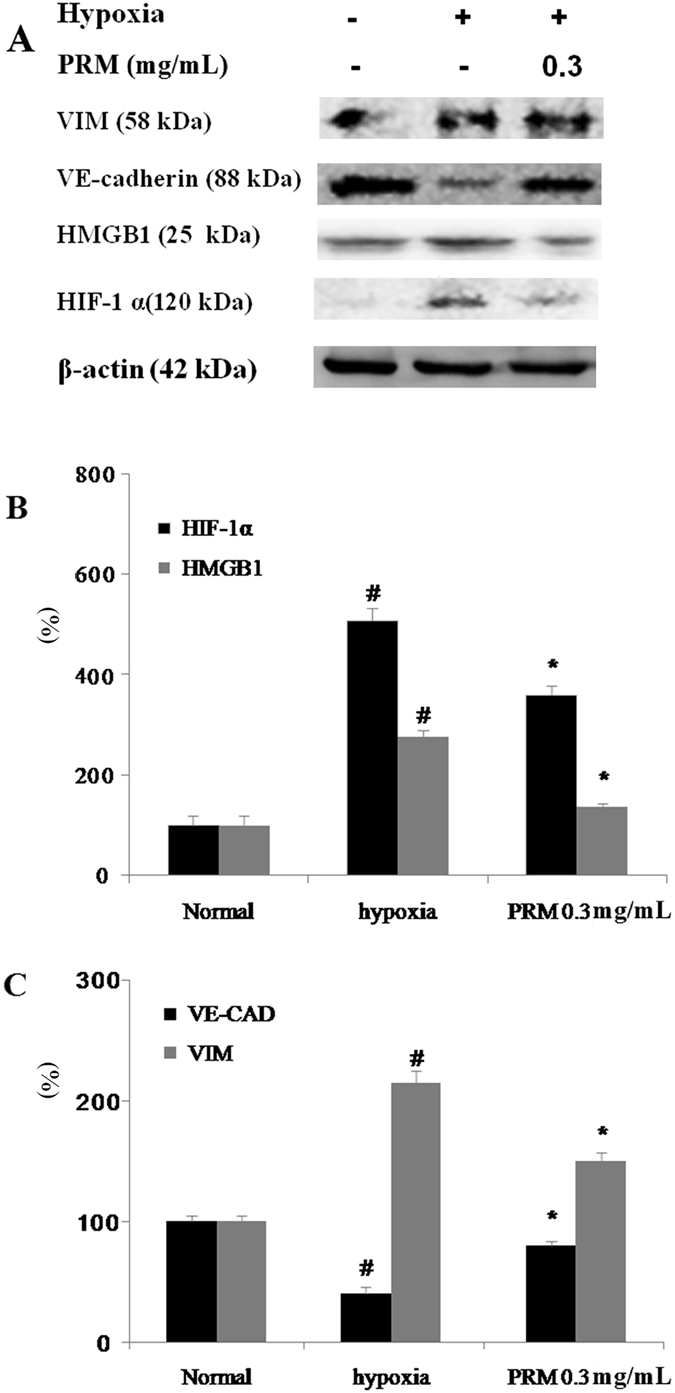
Effects of PRM on EndoMT in HPMECs. (**A–C**) HPMECs were incubated under hypoxic conditions for 48 h. Representative western blots of VE-cadherin, VIM, HMGB1 and HIF-1α. All data are shown as the mean ± S.D., ^#^P < 0.01 vs. the normal group; ^*^P < 0.05, ^**^P < 0.01 vs. the hypoxia stimulated group. Significance was determined by one-way ANOVA followed by Dunnett’s test. The full-length blots of Fig. 3A are presented in Supplementary information 2.2.

When HPMECs were exposed to CoCl_2_ for 48 h, EndoMT was enhanced as demonstrated by a higher expression of the mesenchymal cell marker vimentin and a lower expression of the endothelial cell marker VE-cadherin. However, EndoMT was attenuated when the CoCl_2_-stimulated cells were exposed to 0.3 mg/mL PRM for 48 h. To investigate how PRM might reduce EndoMT, its effects on the expression of HMGB1 and HIF-1α were analyzed by western blot. The results demonstrated that the treatment of CoCl_2_-stimulated HPMECs with PRM attenuated the increase in HMGB1 and HIF-1α expression as shown in Fig. 3B and C.

EndoMT is a newly recognized type of cellular trans-differentiation and is another possible source of myofibroblasts. EndoMT plays an important role in the pathogenesis of IPF; therefore, the inhibition of EndoMT may represent a novel therapeutic target for IPF^21, 22^. Our studies demonstrated that the majority of HPMECs that were treated with PRM were resistant to CoCl_2_-induced EndoMT, whereas PRM-untreated controls were more vulnerable to EndoMT. This finding suggested that PRM reduced CoCl_2_-induced EndoMT, and thus reduced and delayed IPF.

#### Effects of PRM on HLF-1 proliferation

**Figure 4:**
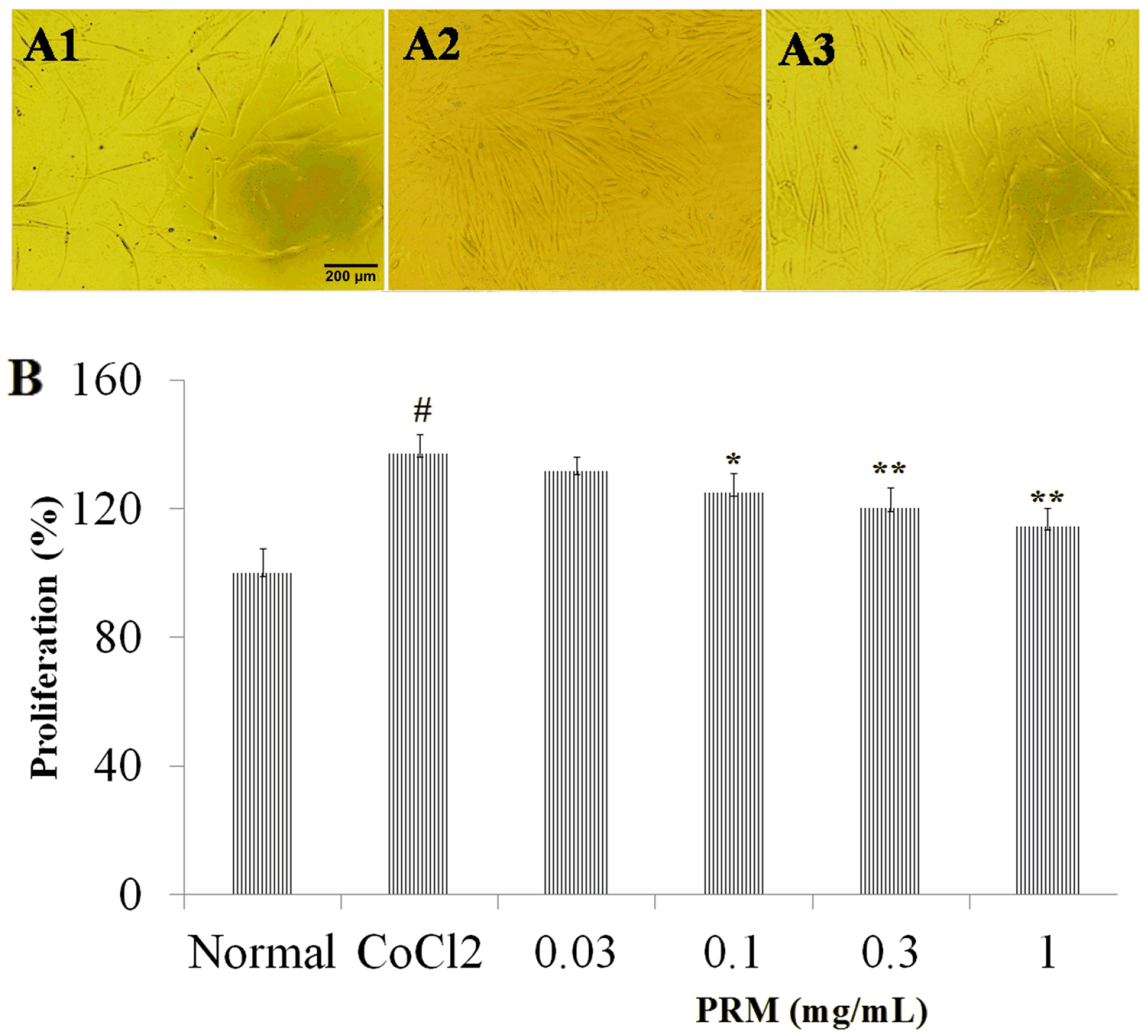
Effects of PRM on cell proliferation in HLF-1. (**A**) Cell proliferation was tested by MTT when HLF-1 cells were treated with CoCl_2_ (100 μM) for 48 h. All data was shown as the mean ± S.D., ^#^P < 0.01 vs. the normal group; ^*^P < 0.05, ^**^P < 0.01 vs. the CoCl_2_ stimulated group. Significance was determined by one-way ANOVA followed by Dunnett’s test.

**Figure 5:**
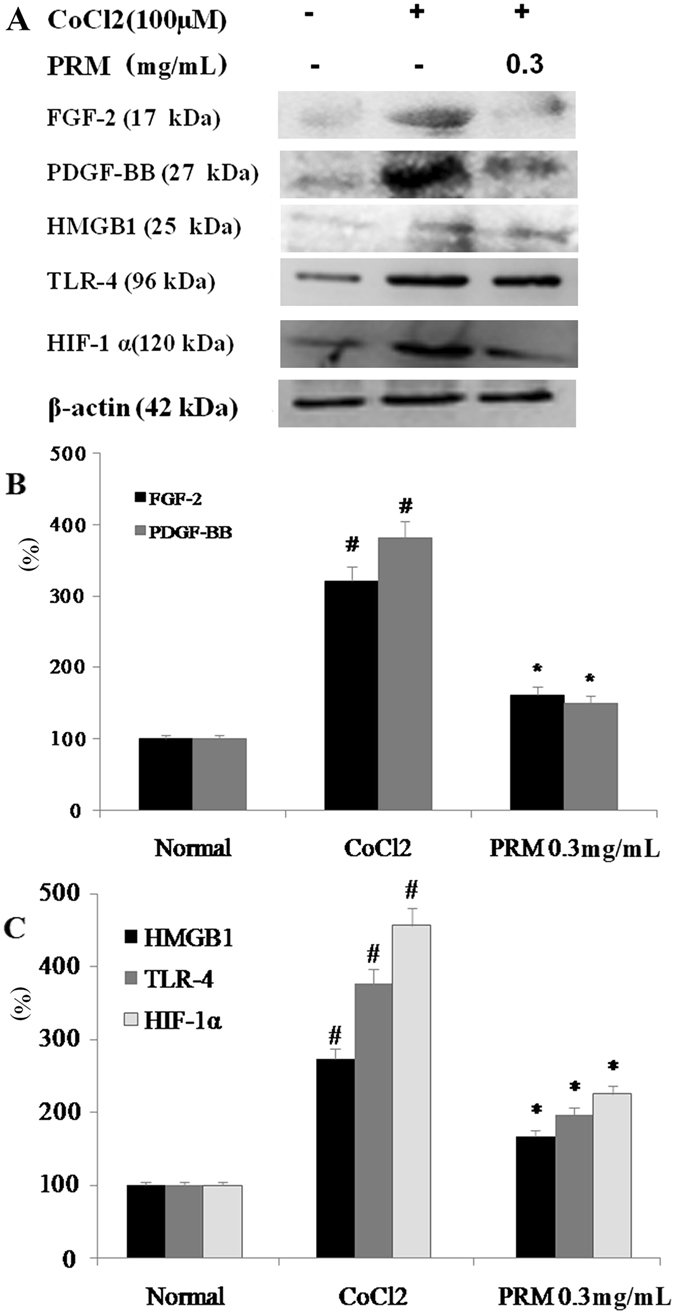
Effects of PRM on HIF-1α, TLR-4, PDGF-BB and FGF-2 expression in CoCl_2_ stimulated HLF-1. (**A**) Representative light microscopic appearance of normal HLF-1 cells (A1), CoCl_2_-treated cells (A2), and cells treated with CoCl_2_ +0.3 mg/mL PRM (A3). (**B** and **C**), HLF-1 cells were incubated with CoCl_2_ (100 μM) for 48 h. HMGB1, HIF-1α, TLR-4, PDGF-BB and FGF-2 expression was analyzed by western blot. The results are reported as percent increase over normal. Data are reported as the mean ± S.D., n = 5. ^#^P < 0.01 vs. the normal group; ^*^P < 0.05, ^**^P < 0.01 *vs*. the CoCl_2_ stimulated group. Significance was determined by one-way ANOVA followed by Dunnett’s test. The full-length blots of Fig. 5A are presented in Supplementary information 2.3.

The HLF-1 cells were continuously exposed to 100 μM CoCl_2_ to mimic the hypoxia that occurs in IPF and induce HLF-1 cell proliferation. HLF-1 proliferation was significantly enhanced in the HLF-1 cells that were treated with 100 μM CoCl_2_ for 72 h. However, the proliferation was attenuated with an IC_50_ of 0.37 mg/mL, when the CoCl_2_-stimulated HLF-1 cells were exposed to 0.1–1.0 mg/mL PRM for 72 h as shown in Fig. 4. FGF2, PDGF, HMGB1, TLR-4 and HIF-1α expression levels were investigated to demonstrate the mechanism of PRM in HLF-1 proliferation. The results showed that CoCl_2_ up-regulated the expression of FGF2, PDGF, HMGB1, TLR-4 and HIF-1α, which resulted in the development of pulmonary fibrosis. However, PRM prevented the cell proliferation that was induced by hypoxia and reduced the expression of FGF2, PDGF, HMGB1, TLR-4 and HIF-1α as shown in Fig. 5.

#### Effects of PRM on pulmonary fibrosis *in vivo*

**Figure 6:**
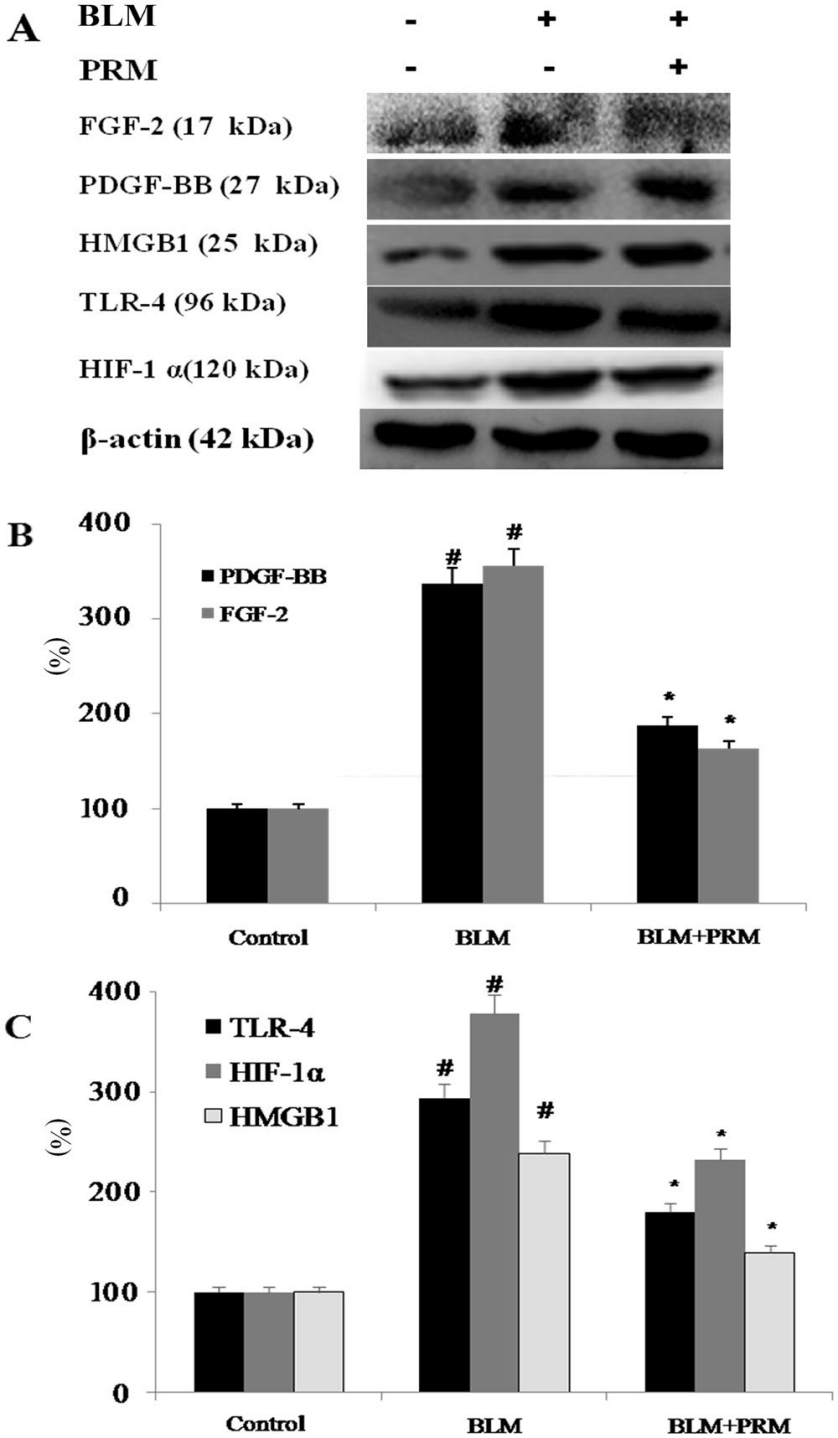
Effects of PRM on FGF-2, PDGF-BB, TLR-4, HMGB1 and HIF-1α expression in PF rats induced by BLM. (**A**) Representative western blots of FGF-2, PDGF, TLR-4, HMGB1 and HIF-1α. (**B** and **C**) FGF-2, PDGF-BB, TLR-4, HMGB1 and HIF-1α expression was analyzed by western blot. The results are reported as percent increase over the sham rats and are shown as the mean ± S.D., ^#^P < 0.01 vs. the sham rats; ^*^P < 0.05, ^**^P < 0.01 vs. the BLM stimulated rats. Significance was determined by one-way ANOVA followed by Dunnett’s test. The full-length blots of Fig. 6A are presented in Supplementary information 2.4.

FGF-2, PDGF, TLR-4, HMGB1 and HIF-1α expressions levels in lung tissues were determined by western blot analyses. The expression of FGF-2, PDGF, TLR-4, HMGB1 and HIF-1α decreased in the PRM-treated rats as shown in Fig. 6A–C. The results of the *in vivo* studies showed that PRM also inhibited EndoMT and fibroblast proliferation in PF rats that was induced by BLM and reduced the expression of FGF-2, PDGF, TLR-4, HMGB1 and HIF-1α.

Pulmonary fibroblast proliferation plays an important role in the progress of IPF. Moreover, PDGF and FGF also play important roles in IPF. The inhibition of pulmonary fibroblast proliferation and PDGF and FGF expression in fibroblasts attenuates fibrosis^23^. The increase in PDGF and FGF expression was observed in hypoxia stimulated HLF-1 cells and PF rats that had been exposed to BLM. Our results showed that PRM treatment reduced PDGF and FGF-2 expression in CoCl_2_-stimulated HPMECs and PF rats that were exposed to BLM.

HIF-1α is a heterodimeric transcription factor that is regulated by oxygen^24^. Hypoxia is a characteristic feature of IPF. Under hypoxic conditions, the HIF-1α protein accumulates^25^. Meanwhile, HMGB1 plays a key role in IPF, especially in the late phase of an acute exacerbation^26^. The elevated expression of HMGB1 and HIF-1α was observed in BLM-treated animals. Reduced serum HMGB-1 levels in IPF patients with an acute exacerbation could improve oxygenation^27^. TLR-4 is the receptor for HMGB1 and plays an important role in age-associated lung diseases, including IPF^28^. Our study showed that PRM reduced HMGB1 and HIF-1α expression in CoCl_2_ stimulated A549 cells, HPMECs and HLF-1, and PF rats that were exposed to BLM. These results support the finding that PRM ameliorates EMT and EndoMT, reduces fibroblast proliferation and reduces fibrosis by modulating HMGB1 and HIF-1α expression. In summary, our findings demonstrated that PRM treatment may have a beneficial effect on PF *in vitro* and *in vivo*.

## Pharmacokinetics of PRM

**Figure 7:**
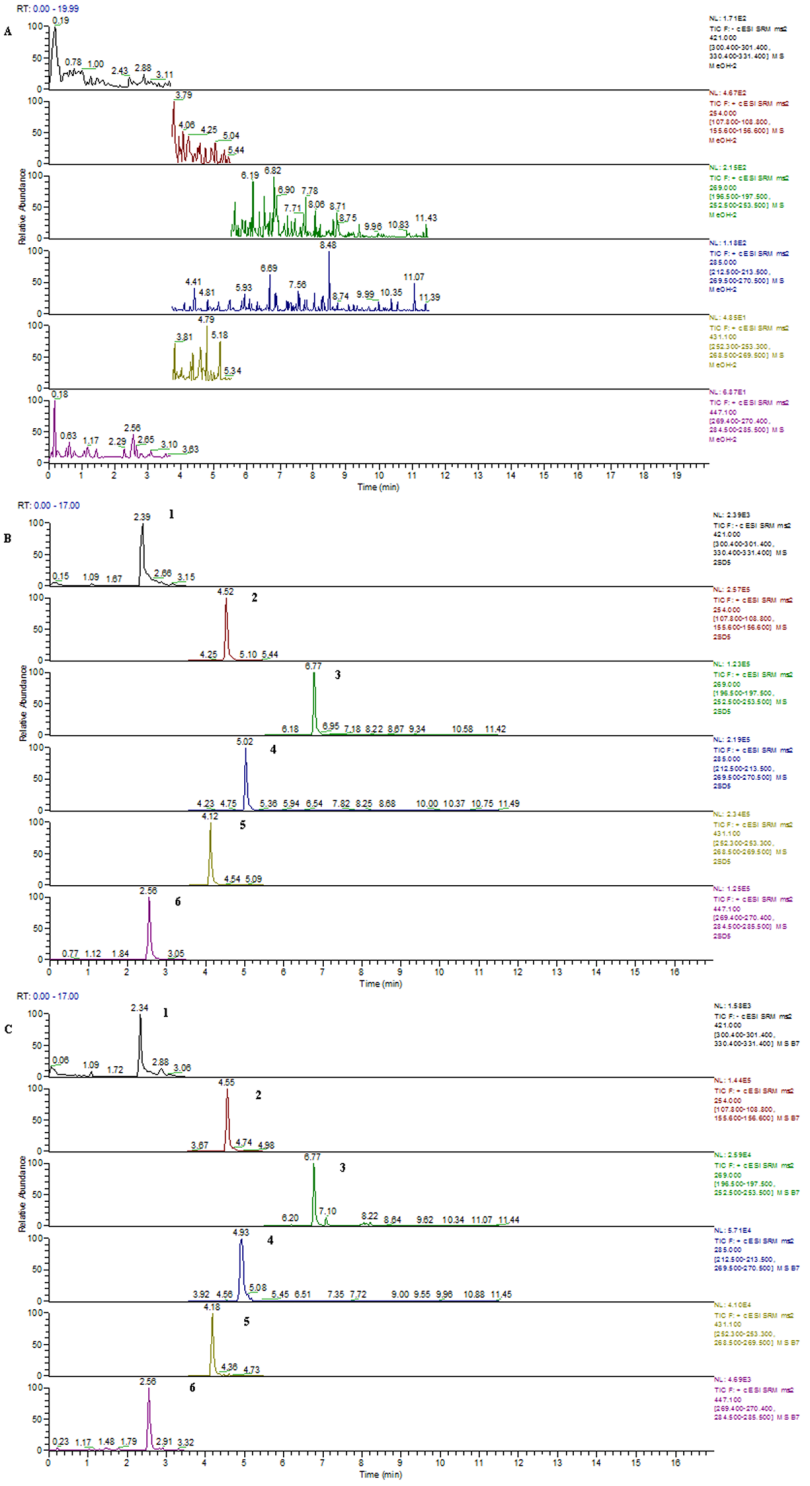
Typical MRM chromatograms of a blank rat plasma sample (**A**), a blank rat plasma spiked with calycosin, calycosin-7-O-glucoside, formononetin, ononin and mangiferin (**B**), and a rat plasma after the oral administration of PRM (**C**). 1: mangiferin, 2: sulfamethoxazole (IS), 3: formononetin, 4: calycosin, 5: ononin, 6: calycosin-7-O-glucoside. The original chromatograms are presented in Supplementary information 3.1–3.3.

A U-HPLC-MS/MS method was developed and validated for the simultaneous determination of calycosin, calycosin-7-O-glucoside, formononetin, ononin and mangiferin in rat plasma. The U-HPLC-MS/MS conditions and the sample preparation method were both optimized for the best results. The retention times were approximately 2.39, 2.56, 4.12, 5.02, 6.77 and 4.12 min for mangiferin, calycosin-7-O-glucoside, ononin, calycosin, formononetin and IS, respectively. Blank plasma samples from six rats were screened and found to be free of endogenous interference for all the analytes because of the high selectivity of the MRM mode. Typical chromatograms of blank plasma, blank plasma spiked with all the analytes and rat plasma samples after the administration of PRM are shown in Fig. 7. The standard calibration curves showed good linearity over the concentration range of 0.2–50 ng/mL for the five analytes with correlation coefficients above 0.99. The regression equations of the analytes are listed in Table 2. In addition, the matrix effect, accuracy, precision, recovery and stability of the method were validated and met the requirements of the “Guidance for Industry: Bioanalytical Method Validation”, which was approved by the FDA.

**Table 2:** The calibration curves of formononetin, ononin, calycosin, calycosin-7-O-glucoside and mangiferin in rat plasma.

Analyte	Regression equation	Correlation coefficient (*r*)
formononetin	y = 7.140 × 10^−2^ x + 2.316 × 10^−2^	0.9978
ononin	y = 6.854 × 10^−2^ x + 5.510 × 10^−2^	0.9943
calycosin	y = 4.014 × 10^−2^ x + 6.446 × 10^−2^	0.9926
calycosin-7-O-glucoside	y = 9.82 × 10^−2^ x + 6.354 × 10^−2^	0.9975
mangiferin	y = 7.185 × 10^−4^ x + 8.95 × 10^−5^	0.9992

**Figure 8:**
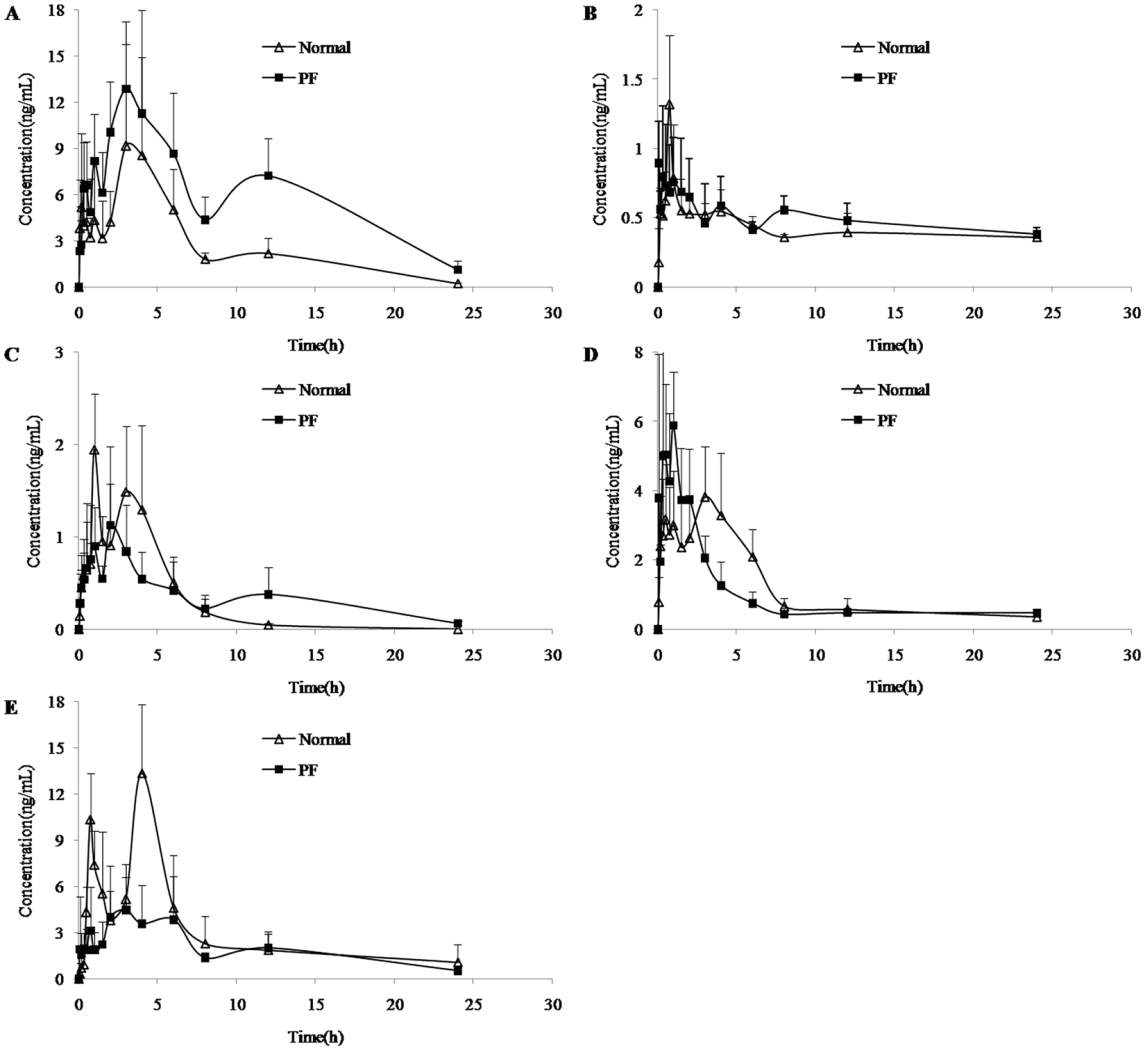
The concentration–time curves of calycosin (**A**), calycosin-7-O-glucoside (**B**), formononetin (**C**), ononin (**D**) and mangiferin (**E**) in rat plasma after the oral administration of PRM in normal and PF groups (n = 6).

The validated U-HPLC-MS/MS method was successfully used to study the pharmacokinetic profiles in rat plasma after the oral administration of PRM. The pharmacokinetic parameters of calycosin, calycosin-7-O-glucoside, formononetin, ononin and mangiferin were calculated by the non-compartmental analysis of plasma concentration *vs*. time data using Winnonlin 5.2.1 (Pharsight Corporation, Mountain View, CA, USA). The mean plasma concentration-time curves of the five analytes are presented in Fig. 8 and the corresponding pharmacokinetic parameters are summarized in Table 3.

**Table 3:** The main pharmacokinetic parameters of calycosin, calycosin-7-O-glucoside, formononetin, ononin and mangiferin after the oral administration of PRM (mean ± SD; *n* = 6).

Parameter	Group	AUC_(0-t)_ (ng × h/mL)	AUC_(0-∞)_ (ng × h/mL)	*C*_max_ (ng/mL)	*T*_1/2_ (h)	*T*_max_ (h)
calycosin	Normal	66.3 ± 31.5	68.1 ± 32.3	11.2 ± 5.7	4.1 ± 1.7	2.8 ± 1.6
	PF	142.9 ± 56.7*	153.5 ± 60.2*	16.3 ± 4.2	6.1 ± 0.9*	2.5 ± 0.8
calycosin-7-O-glucoside	Normal	10.2 ± 2.0	28.2 ± 5.3	1.5 ± 1.0	34.7 ± 10.9	0.6 ± 0.4
	PF	10.3 ± 3.7	26.0 ± 11.7	1.5 ± 0.8	27.5 ± 15.3	1.3 ± 1.1
formononetin	Normal	7.2 ± 3.9	7.7 ± 4.1	2.5 ± 1.8	2.3 ± 1.3	1.3 ± 1.0
	PF	6.6 ± 3.1	8.3 ± 4.1	1.6 ± 0.7	6.1 ± 2.1*	1.9 ± 1.0
ononin	Normal	24.8 ± 8.1	28.2 ± 9.9	4.9 ± 1.2	5.6 ± 3.2	1.4 ± 0.9
	PF	17.1 ± 4.5	18.4 ± 4.8	7.2 ± 2.1	2.4 ± 0.5	0.6 ± 0.4
mangiferin	Normal	75.0 ± 27.3	111.9 ± 47.4	16.3 ± 5.9	17.2 ± 11.3	3.0 ± 1.4
	PF	20.1 ± 7.6*	25.6 ± 7.8*	4.4 ± 2.2*	10.7 ± 5.2	4.8 ± 2.7

^*^ *P* < 0.05 compared with the normal group.

As shown in Fig. 8, calycosin, calycosin-7-O-glucoside, formononetin, ononin and mangiferin were all detected over the limit of quantification, which indicated that the five constituents could be absorbed into the blood after the oral administration of PRM. In addition, the five constituents exhibited different patterns in plasma concentration-time profiles. The peak plasma concentrations (Cmax) of calycosin, ononin and mangiferin were higher than 5.0 ng/mL, while the Cmax values of calycosin-7-O-glucoside and formononetin were low (approximately 1.0–2.0 ng/mL). The main reason for the low plasma concentrations was the low content of 5 analytes in the PRM and the low dosage to the rat. Additionally, the drug metabolism *in vivo* also may result in low plasma concentration. Calycosin-7-O-glucoside could be metabolized to calycosin *in vivo*, so the plasma concentration of calycosin-7-O-glucoside was lower than calycosin, while the content of calycosin-7-O-glucoside in the PRM was higher than calycosin. Therefore, these results suggested that the five constituents may be the effective ingredients in PRM according to the plasma pharmacochemistry^29^. In addition, some studies have reported the biological anticancer effects of calycosin, ononin and mangiferin.

The pharmacokinetic parameters, including AUC_(0-t)_, AUC_(0-∞)_ and *C*_max_, were compared via independent-samples t-tests with SPSS 16.0, while the *T*_1/2_ and *T*_max_ were analyzed with the Mann-Whitney test. As shown in Table 3, there were significant differences (P < 0.05) in the pharmacokinetic parameters for calycosin, formononetin and mangiferin in the normal and PF rats; however, no significant difference was observed for ononin and calycosin-7-O-glucoside. Compared to the normal rats, the AUC_(0-t)_, AUC_(0-∞)_ and T_1/2_ for calycosin were significantly increased (P < 0.05) in the PF rats, and the significant increase in AUC might indicate that more calycosin was absorbed in the PF rats. In contrast, the AUC_(0-t)_ and AUC_(0-∞)_ of mangiferin remarkably decreased in the PF rats. Moreover, the T_1/2_ of calycosin and formononetin were prolonged in PF rats compared to normal rats.

Briefly, there were distinct differences in the pharmacokinetic profiles of PRM in the normal and PF rats. Nevertheless, the reasonable mechanisms of these changes have not been completely explained. The possible explanation for these differences appears to involve the physiological changes in the PF rats, which may affect the pharmacokinetic behavior of PRM. For example, PF may induce a gastroesophageal reflux^30^, which would most likely influence the absorption of PRM. More studies will be conducted to discover the exact reasons that underlie these effects.

## Conclusions

In this paper, we systematically studied the pharmacodynamics and pharmacokinetics of PRM. The pharmacodynamic results demonstrated that PRM could attenuate EMT and EndoMT in CoCl_2_-stimulated A549 cells and HPMECs, respectively, and could inhibit the proliferation of HLF-1 at an IC_50_ of 0.37 mg/mL *in vitro*. In addition, PRM treatment ameliorated BLM-induced PF and reduced the expression of FGF-2, PDGF-BB, TLR-4, HMGB1 and HIF-1α *in vivo*. In the pharmacokinetic study, an accurate and sensitive U-HPLC-MS/MS method was developed and validated for the simultaneous determination of calycosin, calycosin-7-O-glucoside, formononetin, ononin and mangiferin in rat plasma for the first time; then, this method was successfully applied to the comparative pharmacokinetic study of PRM in normal and PF rats. The five constituents of PRM could be absorbed into the blood after the oral administration and exhibited different patterns in plasma concentration-time profiles in normal and PF rats. These results suggest that the five constituents may be the effective ingredients in PRM according to the plasma pharmacochemistry.

In summary, PRM exhibited a satisfactory pharmacodynamic and pharmacokinetic performance which highlights PRM as a potential multi-target oral drug for the treatment of PF.

## Electronic supplementary material

Supplementary information for No. SREP-16-22940
