# Supplementary material for: Pharmacodynamic and pharmacokinetic assessment of pulmonary rehabilitation mixture for the treatment of pulmonary fibrosis
Source: Sci Rep. 2017 Jun 14;7:3458. doi: 10.1038/s41598-017-02774-1 (PMC5471221; doi:10.1038/s41598-017-02774-1)
Supplement: Supplementary file 1 — Supplementary information for No. SREP-16-22940 [file 41598_2017_2774_MOESM1_ESM.doc]

**[Supplementary information](http://www.nature.com/srep/publish/guidelines#supplementary-info) for No. SREP-16-22940**

Title: Pharmacodynamic and pharmacokinetic assessment of pulmonary rehabilitation mixture for the treatment of pulmonary fibrosis

Juanjuan Zhao^1^, Yan Ren^1^, Yubei Qu, Wanglin Jiang^*^, Changjun Lv^*^

**Affiliation**

School of Pharmaceutical Sciences, Binzhou Medical University, Yantai, PR China

**Corresponding author**

Prof. Changjun Lv, School of Pharmaceutical Sciences, Binzhou Medical University, Yantai, 264003, P.R. China.

Tel., +86-535-6913375

Fax, +86-535-6913375

E-mail: Lucky_lcj@sina.com(C.Lv), jwl518@163.com (W. Jiang),

[The supplementary information](http://www.nature.com/srep/publish/guidelines#supplementary-info) include three parts: the detail information on how to quality control of the PRM, full-length gels and blots used in the main figures, the original chromatograms of Figure 7 and the mass spectrum of calycosin, calycosin-7-O-glucoside, formononetin, ononin,mangiferin and sulfamethoxazole.

**1. The detail information on how to quality control of the PRM**

To control the quality of PRM, the chemical fingerprint and the contents of 6 ingredients were determined by high performance liquid chromatographic method (HPLC). The assay was performed on a Shimadzu (Japan) LC system. Liquid chromatographic separation was achieved on Apollo C_18_ column (250 × 4.6 mm, 5 μm). The mobile phase consisted of acetonitrile (A) and 0.1% formic acid aqueous (B). The gradient program was as follows: 0-20min, linear gradient 3-19% A; 20-30 min isocratic elution with 19% A; 30-57 min linear gradient 19-60% A; 57-65 min linear gradient 60-90% A. Chromatography was performed at 35 ºC. The flow rate was 1.0 ml/min and aliquots of 10 µl were injected. The UV detection wavelength was set at 254 nm.

Standard stock solutions containing 6 analytes were prepared and diluted to appropriate concentrations for the method validation. The linearity, precision and accuracy all satisfied with basic requirements. Then the method was applied to determine the fingerprint and 6 ingredients in the PRM for quality control. The fingerprint of PRM was shown in supplementary Fig.1 and the contents of 6 ingredients were summarized in supplementary table 1.

Supplementary table 1 The original herb and contents of ingredients in the PRM

| Original herb | Ingredient | Retention time | content(µg/g) |
| --- | --- | --- | --- |
| *Anemarrhenae Rhizoma* | mangiferin | 21.88 | 296.7 |
| *Rhizoma*. *Astragali Radix* | calycosin-7-O-glucoside | 28.42 | 220.4 |
| *Glycyrrhizae Radix et Rhizoma* | liquiritin | 30.44 | 413.7 |
| *Rhizoma*. *Astragali Radix* | ononin | 43 | 173.6 |
| *Rhizoma*. *Astragali Radix* | calycosin | 46.22 | 124.4 |
| *Rhizoma*. *Astragali Radix* | formononetin | 53.67 | 50.8 |


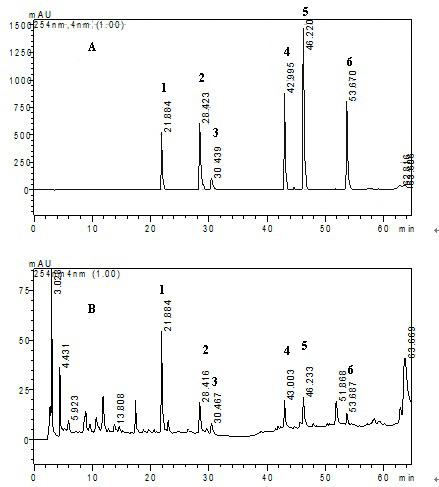


Supplementary Fig. 1. HPLC fingerprint of mix standards (A) and PRM (B). 1: mangiferin, 2: calycosin-7-O-glucoside, 3: liquiritin, 4: ononin, 5: calycosin, 6: formononetin

**2. Full-length gels and blots**

**2.1 Full-length blots/gels for Fig 2B**

HMGB1 (25 kDa)


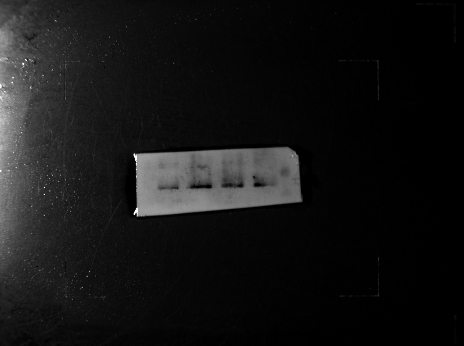


TLR-4 (96 kDa)


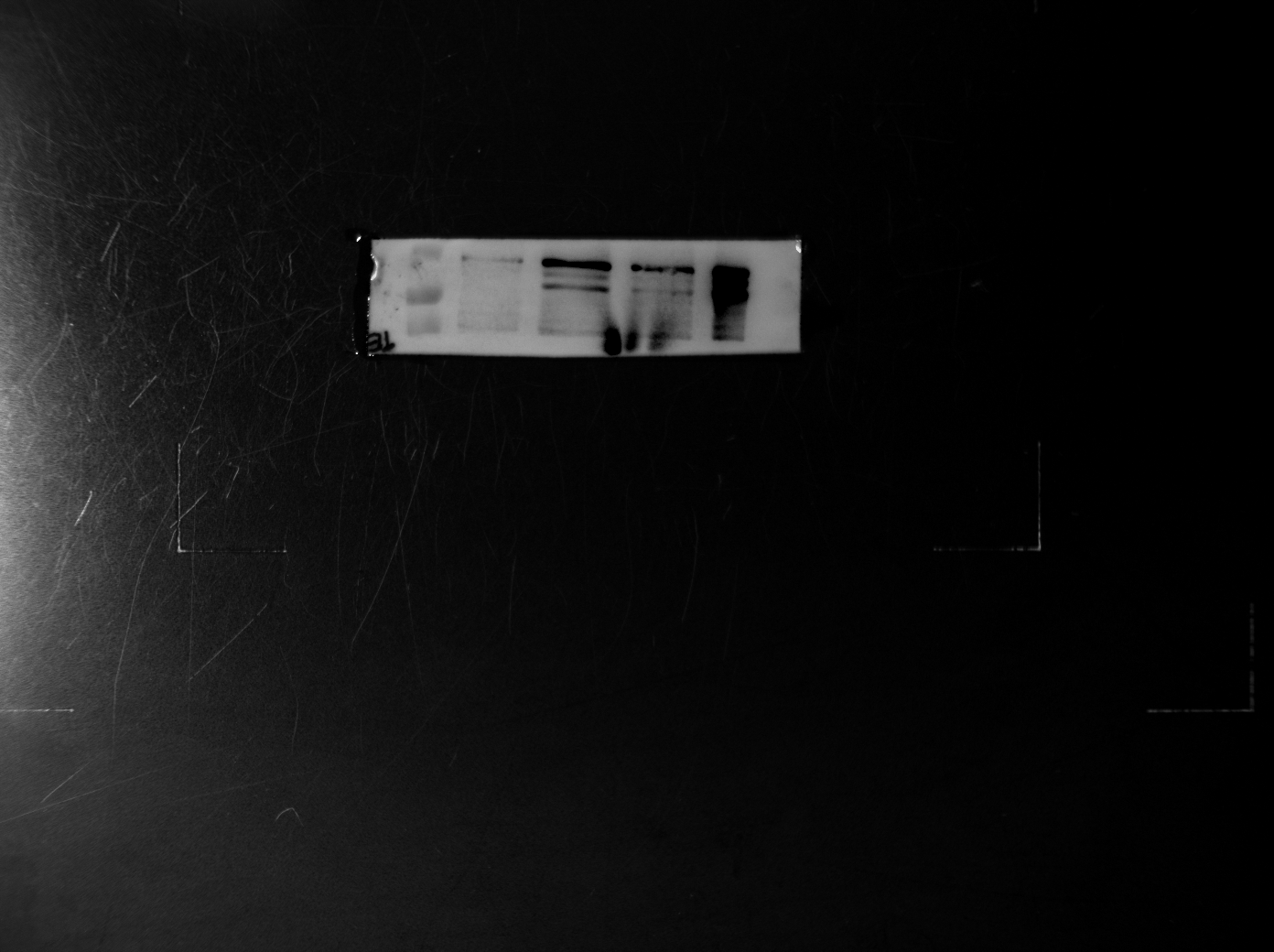


HIF-1α (120 kDa)


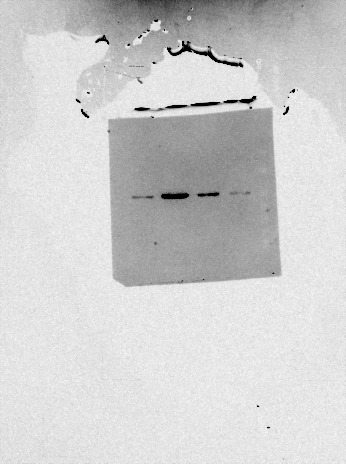


β-actin (42 kDa)

**
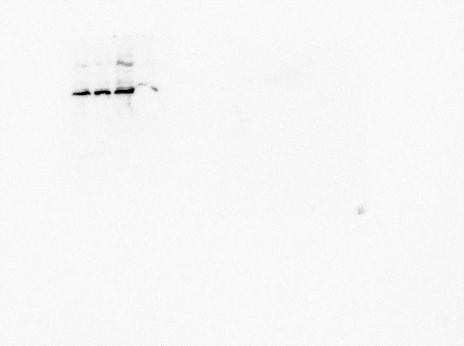
**

**2.2 Full-length blots/gels for Fig 3A**

VIM (58 kDa)


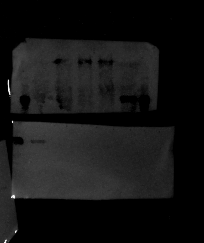


VE-cadherin (88 kDa)


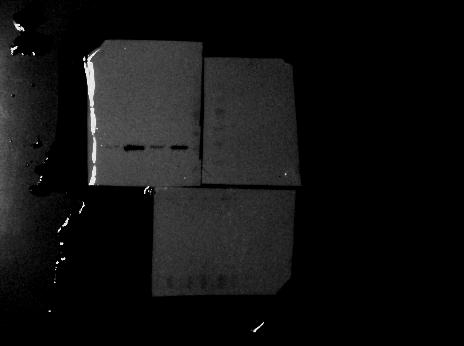


HMGB1 (25 kDa)


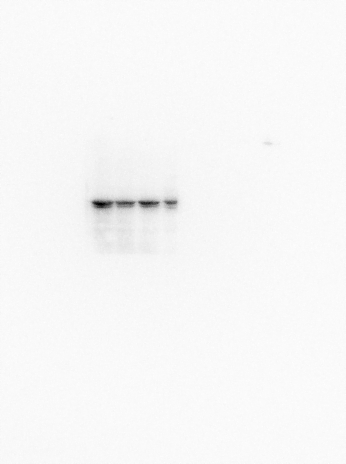


HIF-1α (42 kDa)


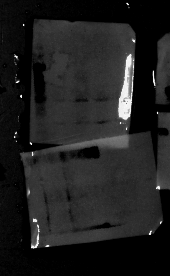


β-actin (42 kDa)

**
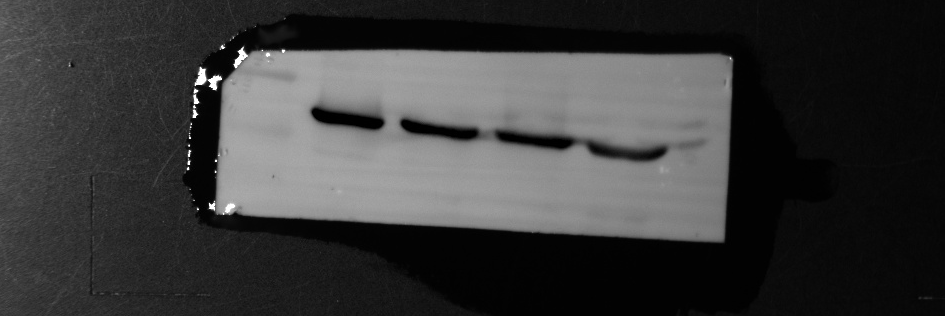
**

**2.3 Full-length blots/gels for Fig 5A**

FGF-2 (17 kDa)


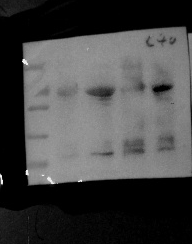


PDGF-BB (27kDa)


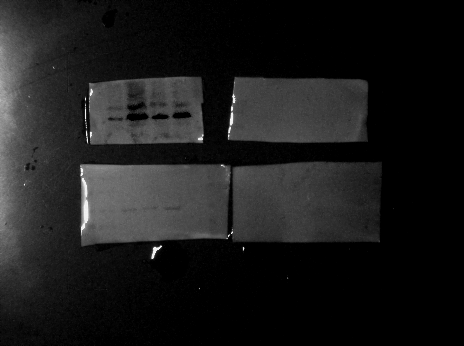


HMGB1 (25kDa)


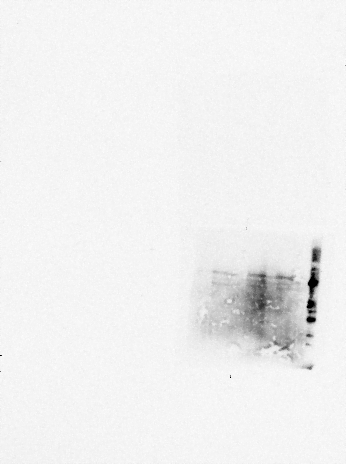


TLR-4 (96 kDa)


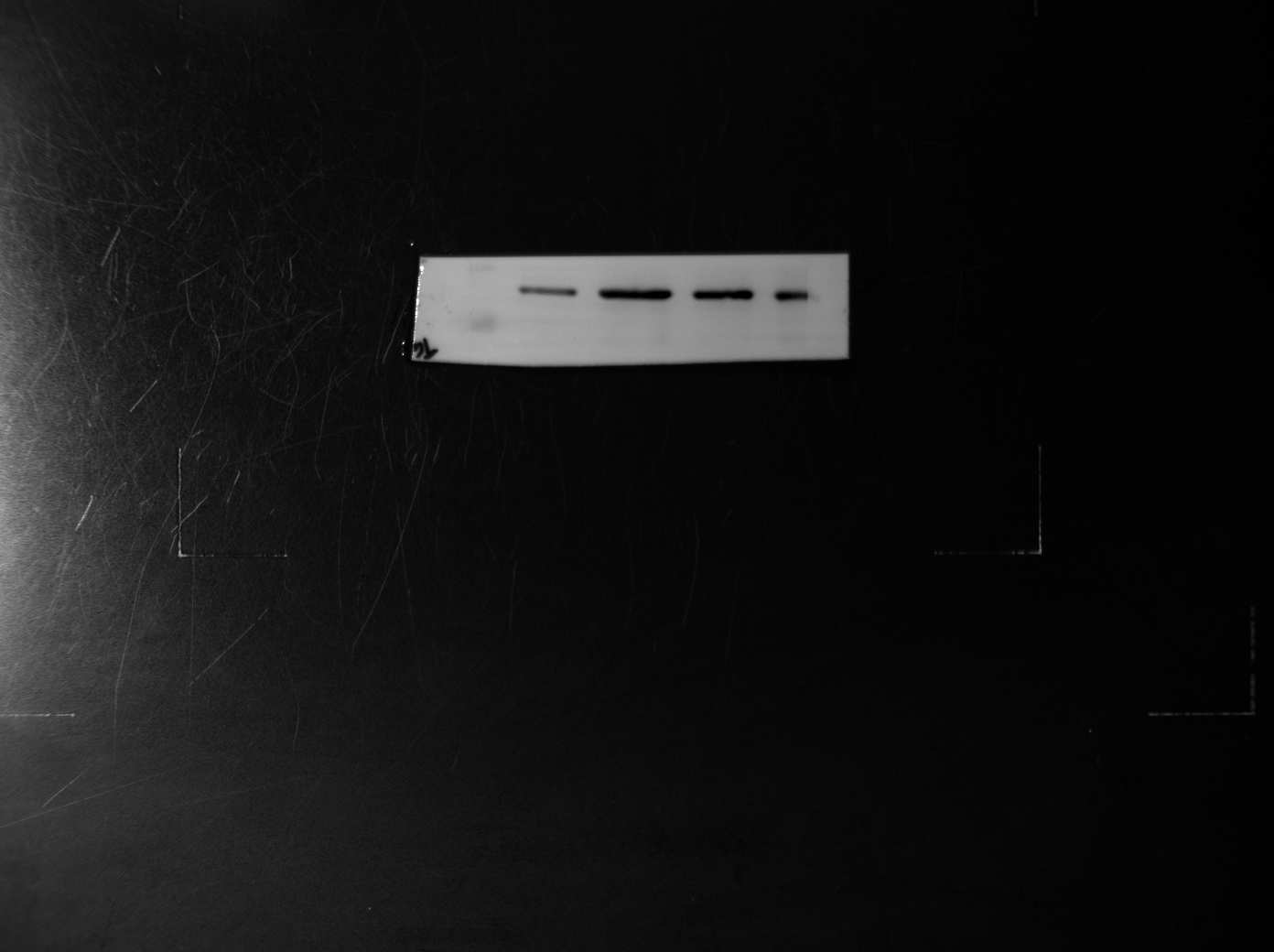


HIF-1α (120 kDa)


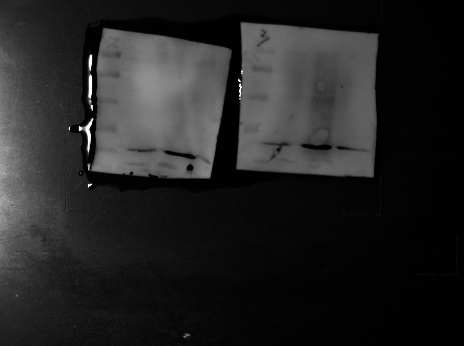


β-actin (42 kDa)

**
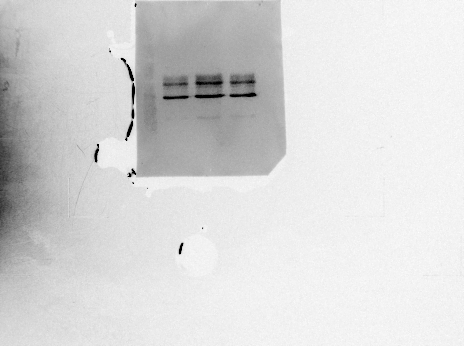
**

**2.4 Full-length blots/gels for Fig 6A**

FGF-2 (17 kDa)


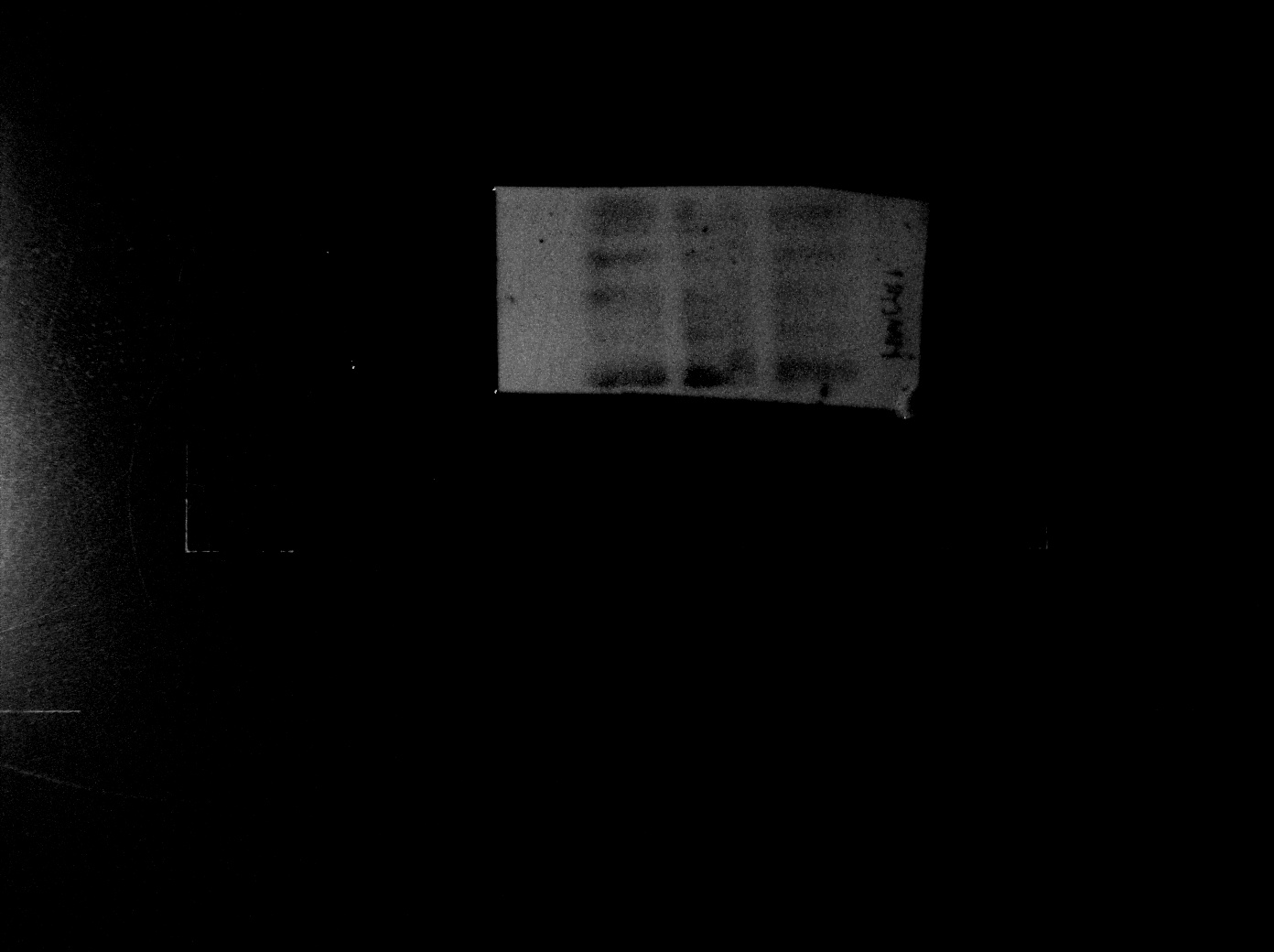


PDGF-BB (27kDa)


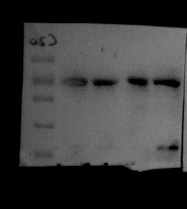


HMGB1 (25kDa)


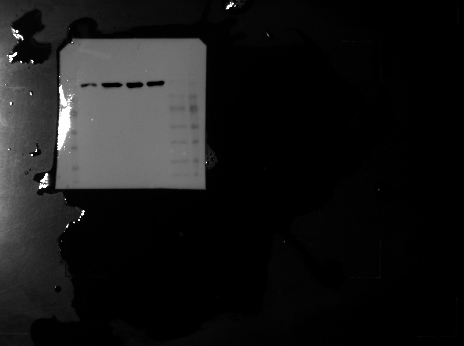


TLR-4 (96 kDa)


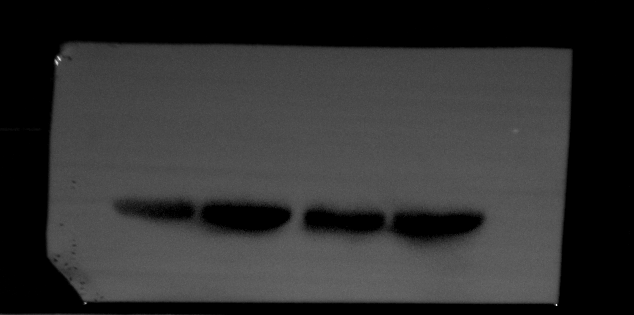


HIF-1α (120 kDa)


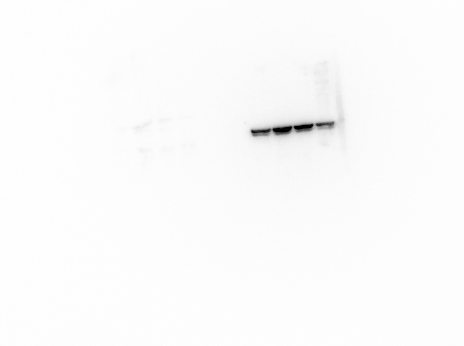


β-actin (42 kDa)


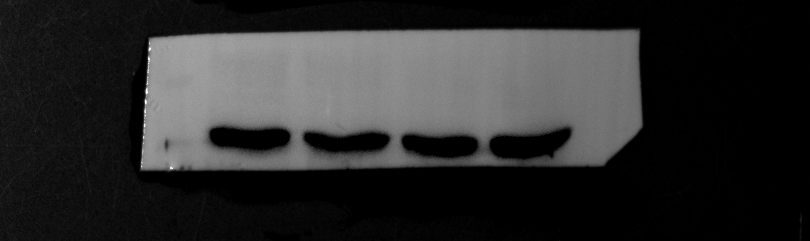


**3. The original chromatograms of Figure 7**

**3.1 Figure 7A**

**3.2 Figure 7B**

**3.3 Figure 7C**

**4. The mass spectrum of six analytes**

Calycosin

Calycosin-7-O-glucoside

Formononetin

Ononin

Mangiferin

Sulfamethoxazole
